# A functional dissociation of face-, body- and scene-selective brain areas based on their response to moving and static stimuli

**DOI:** 10.1038/s41598-019-44663-9

**Published:** 2019-06-03

**Authors:** David Pitcher, Geena Ianni, Leslie G. Ungerleider

**Affiliations:** 1 Department of Psychology, University of York, Heslington York, YO105DD UK; 2 Weill Cornell/Rockefeller/Sloan Kettering Tri-Institutional MD-PhD Program, New York, 10065 USA; 3 Section on Neurocircuitry, Laboratory of Brain and Cognition, National Institute of Mental Health, Bethesda, MD 20892 USA

**Keywords:** Perception, Object vision

## Abstract

The human brain contains areas that respond selectively to faces, bodies and scenes. Neuroimaging studies have shown that a subset of these areas preferentially respond more to moving than static stimuli, but the reasons for this functional dissociation remain unclear. In the present study, we simultaneously mapped the responses to motion in face-, body- and scene-selective areas in the right hemisphere using moving and static stimuli. Participants (N = 22) were scanned using functional magnetic resonance imaging (fMRI) while viewing videos containing bodies, faces, objects, scenes or scrambled objects, and static pictures from the beginning, middle and end of each video. Results demonstrated that lateral areas, including face-selective areas in the posterior and anterior superior temporal sulcus (STS), the extrastriate body area (EBA) and the occipital place area (OPA) responded more to moving than static stimuli. By contrast, there was no difference between the response to moving and static stimuli in ventral and medial category-selective areas, including the fusiform face area (FFA), occipital face area (OFA), amygdala, fusiform body area (FBA), retrosplenial complex (RSC) and parahippocampal place area (PPA). This functional dissociation between lateral and ventral/medial brain areas that respond selectively to different visual categories suggests that face-, body- and scene-selective networks may be functionally organized along a common dimension.

## Introduction

Neuroimaging studies report multiple areas in the human brain that selectively respond to faces, bodies and scenes. The existence of multiple areas for these three categories of visual stimuli has led to proposals that face, body and scene perception is performed in specialized brain networks^1–3^. While these network models have been highly influential, we still lack a full understanding of how these networks function, and of the specific cognitive operations performed in each of the component brain areas. In the current study, we sought to functionally dissociate the components of these face-, body- and scene-selective networks based on their response to moving and static stimuli. Our aim was to look for functional dissociations that are common across category-selective brain areas to better understand how the networks that process these categories are functionally organized in the human brain.

Face-, body- and scene-selective areas are found on the ventral, medial, and lateral surfaces of the occipital and temporal cortex. Ventral areas include the fusiform face area (FFA)^4,5^, occipital face area (OFA)^6^, fusiform body area (FBA)^7^, and parahippocampal place area (PPA)^8^. Medial areas include the scene-selective retrosplenial complex (RSC^9^) and face-selective voxels in the amygdala^10^. Lateral areas include face-selective regions in the posterior superior temporal sulcus (pSTS)^11,12^ and anterior superior temporal sulcus (aSTS)^13^, the extrastriate body area (EBA)^14^ and the occipital place area (OPA)^15,16^. Network models propose that the lateral areas represent more primitive, local, and stimulus-driven components, while ventral areas represent invariant and global features linked to the subjective percept^1–3,17^. Prior studies have provided evidence that supports this hierarchical division between ventral and lateral areas in the face network^18–20^, the body network^21,22^ and the scene network^23^. However, these hierarchical dissociations between lateral and ventral areas tend to focus on cognitive operations such as within-category exemplar recognition and do not fully account for how these areas differentially process moving stimuli.

The motion-selective area, V5/MT, is located on the lateral surface, close to the intersection of the ascending limb of the inferior temporal sulcus and the lateral occipital sulcus^24^. Areas surrounding V5/MT in the lateral occipitotemporal cortex (LOTC) have also been shown to represent different aspects of action perception^25^. These include perception of bodies^26^, tools^27^ and action observation^28^. Anterior to human V5/MT is the superior temporal sulcus (STS), which also responds to a wide range of moving biological stimuli. These include faces^12,29,30^, point-light walkers^31,32^, bodies^33^ and the perception of goal-directed actions^34–37^ By contrast, the strong preference for moving stimuli on the lateral surface is less, or absent, in category-selective regions on the ventral surface. Prior studies have demonstrated a greater response for moving stimuli in lateral than ventral brain areas, using different stimuli including faces^30,38–40^, bodies^41^ and scenes^42^.

To date these studies have largely focused on a single visual category (e.g. faces, bodies or scenes) and have not simultaneously compared the response to moving and static stimuli across multiple object categories in the same group of experimental participants. In addition, the response to moving and static stimuli in the place-selective retrosplenial complex (RSC), as well as in the face-selective voxels of the amygdala is unknown. In the current study, participants (N = 22) were scanned using fMRI at 7 Tesla while viewing 3 second videos containing bodies, faces, objects, scenes and scrambled objects, or static pictures taken from the beginning, middle and end of each video. Our aim was to simultaneously measure the differential response to moving and static stimuli across all face-, body- and scene-selective areas in the brain to establish differences and similarities across different visual categories.

## Results

### Identifying ROIs

Face, body and scene-selective areas were identified using short videos displaying bodies, faces, objects, scenes, and scrambled objects^40^. We were able to identify the necessary ten ROIs in the right hemisphere of eighteen of the twenty-two participants. Face-selective ROIs (identified using a contrast of faces > objects) included the FFA, OFA, pSTS, aSTS and face-selective voxels in the amygdala (four participants did not show any face-selective voxels in the amygdala). The two body-selective ROIs (identified using a contrast of bodies > objects) were EBA and FBA. Scene-selective ROIs (identified using a contrast of scenes > objects) included PPA, RSC and OPA. All ROIs were identified based on the activation of peak voxels in the relevant brain areas identified by prior studies^4,6–9,14,16,40^. We selected all contiguous voxels for each ROI.

In the left hemisphere, we were unable to identify the necessary ROIs (FFA *N* = 22; OFA *N* = 18; pSTS *N* = 18; aSTS *N* = 11; amygdala *N* = 11; EBA *N* = 22; FBA *N* = 15; PPA *N* = 22; RSC *N* = 20; OPA *N* = 15) in the same 22 participants. This difference between category-selective regions across hemispheres has been reported in prior face-processing studies^4,19,40,43,44^. Consequently, our subsequent analysis focused only on data from the right hemisphere (data from the left hemisphere ROIs are included in supplemental figures).

### ROI response profiles for face-, body- and scene-selective areas

We calculated the percent signal change (PSC) data for moving and static stimuli from all five categories (bodies, faces, objects, scenes and scrambled objects) in category-selective ROIs. To establish which face-, body- and scene-selective ROIs showed a differential response to moving and static stimuli, we then entered the data into a 2 (motion: moving/static) by 5 (stimulus: bodies/faces/objects/scenes/scrambled objects) by 10 (ROI: FFA/OFA/pSTS/aSTS/amygdala/EBA/FBA/PPA/RSC/OPA) Greenhouse-Geisser corrected repeated-measures analysis of variance (ANOVA). We found significant main effects of stimulus (F (4,68) = 44, p < 0.001; partial η ^2^ = 0.733) and ROI (F (9,153) = 35, p < 0.001; partial η ^2^ = 0.672) but not of motion (F (1,17) = 0.3, p = 0.57; partial η ^2^ = 0.021). Stimulus and ROI combined in a significant interaction (F (36,612) = 67, p < 0.001; partial η ^2^ = 0.793) but there was no significant interaction between stimulus and motion (F (4,68) = 1.1, p = 0.36; partial η ^2^ = 0.062) or between motion and ROI (F (9,153) = 2.1, p = 0.12; partial η ^2^ = 0.112). Motion, stimulus and ROI combined in a significant three-way interaction (F (36,612) = 4.9, p < 0.001; partial η ^2^ = 0.233).

### Face-selective ROIs

**Figure 1:**
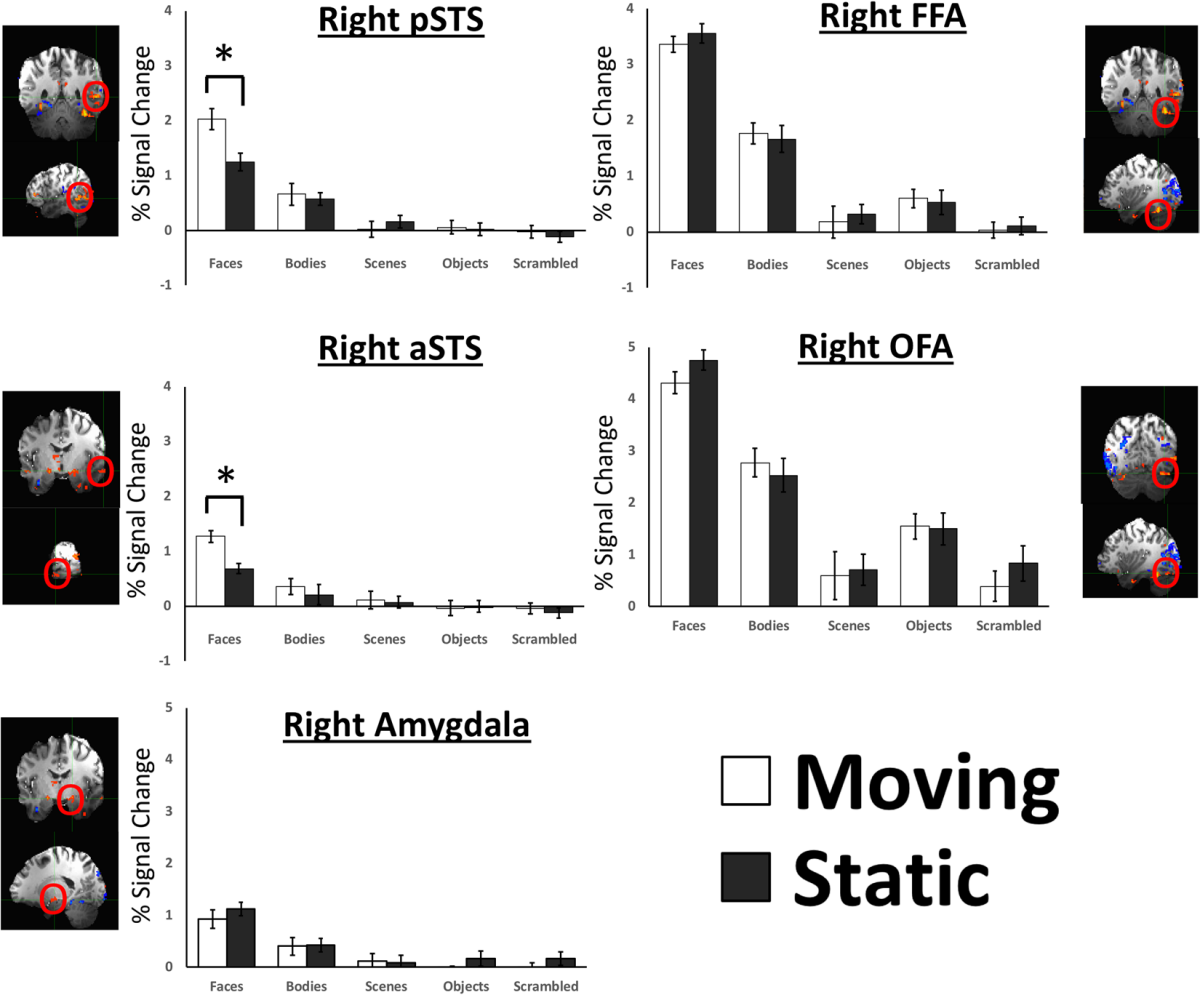
Percent signal change data for the moving and static stimuli from all five categories (faces, bodies, scenes, objects and scrambled objects) in face-selective ROIs. The rpSTS and raSTS, ROIs on the lateral surface showed a significantly greater response to moving faces than to static faces (*denotes significant effects in Bonferroni corrected tests p < 0.001).

The neural response to the moving and static face, body, scene, object and scrambled object stimuli in face-selective ROIs is shown in Fig. 1. To further understand what factors were driving the significant effects, we performed a separate ANOVA on the face-selective ROIs. A 2 (motion: moving/static) by 5 (stimulus: bodies/faces/objects/scenes/scrambled objects) by 5 (ROI: FFA/OFA/pSTS/aSTS/amygdala) Greenhouse-Geisser corrected repeated-measures ANOVA found main effects of stimulus (F (4, 68) = 89.5; p < 0.0001; partial η ^2^ = 0.84) and ROI (F (4, 68) = 95.5; p < 0.0001; partial η ^2^ = 0.85) but not of motion (F (1, 17) = 0; p = 0.997; partial η ^2^ = 0.0). There was a significant two-way interaction between stimulus and ROI (F (16, 272) = 41.5; p < 0.00001; partial η ^2^ = 0.71) but not between motion and stimulus (F (4, 68) = 0.82; p = 0.52; partial η ^2^ = 0.046) or ROI and motion (F (4, 68) = 1.1; p = 0.38; partial η ^2^ = 0.58). The three-way interaction between motion, stimulus and ROI was significant (F (16, 272) = 5; p < 0.0001; partial η ^2^ = 0.226). To further understand what factors were driving the significant effects, we then performed separate two-way ANOVAs on each face-selective ROI.

#### rFFA

A 2 (motion) × 5 (stimulus) repeated-measures ANOVA (Greenhouse-Geisser corrected) showed a main effect of stimulus (*F* (4, 68) = 101, *p < *0.001; partial η ^2^ = 0.856), with a significantly greater response to faces than to any other stimulus (Bonferroni corrected post-hoc comparisons, all *p*’s < 0.0001). The response to bodies was also significantly greater than the response to other categories (Bonferroni corrected post-hoc comparisons, all *p*’s < 0.02). There was no main effect of motion (*F* (1, 17) = 0.7, *p* = 0.8; partial η ^2^ = 0.004) and the interaction between motion and stimulus was not significant (*F* (4, 68) = 0.8, *p* = 0.5; partial η ^2^ = 0.045).

#### rOFA

A 2 (motion) × 5 (stimulus) repeated-measures ANOVA (Greenhouse-Geisser corrected) showed a main effect of stimulus (*F* (4, 68) = 72, *p* < 0.0001; partial η ^2^ = 0.809), with a significantly greater response to faces than to any other stimulus (Bonferroni corrected post-hoc comparisons, all *p*’s < 0.0001). There was no main effect of motion (*F* (1, 17) = 0.25, *p* = 0.6; partial η ^2^ = 0.014) and there was no interaction between motion and stimulus (*F* (4, 68) = 2.6, *p* = 0.1; partial η ^2^ = 0.085).

#### rpSTS

A 2 (motion) × 5 (stimulus) repeated-measures ANOVA (Greenhouse-Geisser corrected) showed main effects of stimulus (*F* (4, 68) = 41, *p* < 0.001; partial η ^2^ = 0.784), with larger responses to faces than all other stimuli (Bonferroni corrected post-hoc comparisons, all *p*’s < 0.001). There was also a larger response to bodies than to scenes, objects and scrambled objects (Bonferroni corrected post-hoc comparisons, all *p*’s < 0.016). The main effect of motion was not significant (*F* (1, 17) = 1.8, *p* = 0.2; partial η ^2^ = 0.095). Motion and stimulus combined in a significant interaction (*F* (4, 68) = 7, p < 0.001; partial η ^2^ = 0.294). Moving faces produced a larger response than static faces in Bonferroni corrected post-hoc comparisons (*p* < 0.001). No other post-hoc tests approached significance (*p* > 0.5).

#### raSTS

A 2 (motion) × 5 (stimulus) repeated-measures ANOVA (Greenhouse-Geisser corrected) showed a significant main effect of stimulus (*F* (4, 68) = 18, *p* < 0.001; partial η ^2^ = 0.623), with a significantly greater response to faces than any other stimulus (Bonferroni corrected post-hoc comparisons, all p’s < 0.001). There was no main effect of motion (*F* (1, 17) = 2.0, *p* = 0.17; partial η ^2^ = 0.16). Motion and stimulus combined in a significant interaction (*F* (4, 68) = 17.1, *p* < 0.001; partial η ^2^ = 0.213). Moving faces produced a larger response than static faces in Bonferroni corrected post-hoc comparisons (*p* = 0.01). No other post-hoc tests approached significance (*p* > 0.4).

#### Amygdala

A 2 (motion) × 5 (stimulus) repeated-measures ANOVA (Greenhouse-Geisser corrected) showed a main effect of stimulus (*F* (4, 68) = 22, *p* < 0.0001; partial η ^2^ = 0.567), with a significantly greater response to faces than other categories (Bonferroni corrected post-hoc comparisons, all *p*’s < 0.001). There was no main effect of motion (*F* (1, 17) = 0.7, *p* = 0.4; partial η ^2^ = 0.04). The interaction between motion and stimulus was not significant (*F* (4, 68) = 0.8, *p* = 0.5; partial η ^2^ = 0.045).

### Body-selective ROIs

**Figure 2:**
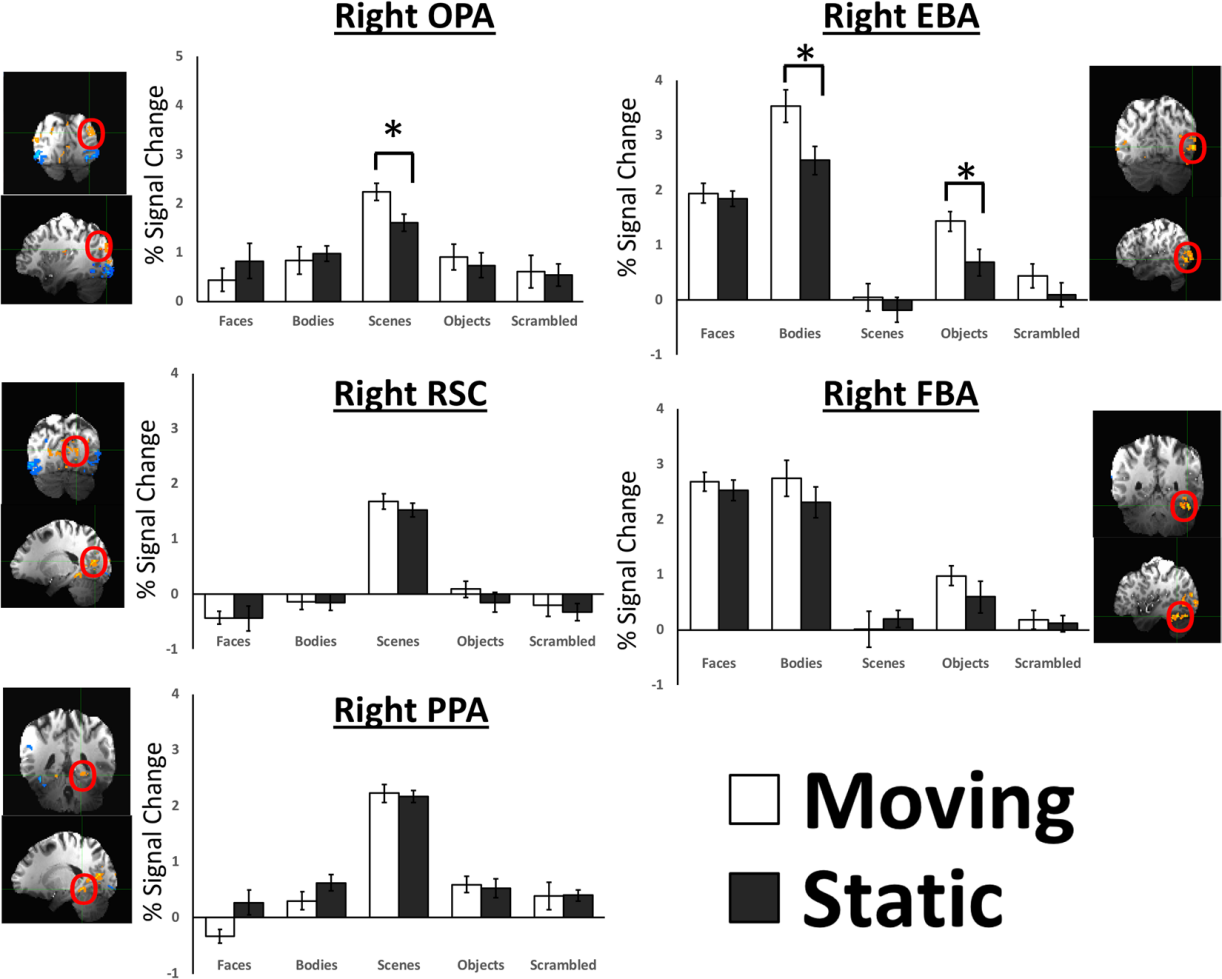
Percent signal change data for the moving and static stimuli from all five categories (faces, bodies, scenes, objects and scrambled objects) in body-selective (right) and scene-selective (left) ROIs. The rEBA and rOPA ROIs on the lateral surface showed a significantly greater response to moving bodies and scenes, respectively, than to static bodies and scenes (*denotes significant effects in Bonferroni corrected tests p < 0.05).

The neural response to the moving and static face, body, scene, object and scrambled object stimuli in body-selective ROIs is shown in Fig. 2 (right panels). For body-selective ROIs a 2 (motion: moving/static) by 5 (stimulus: bodies/faces/objects/scenes/scrambled objects) by 2 (ROI: EBA/FBA) Greenhouse-Geisser corrected repeated-measures ANOVA found main effects of motion (F (1, 17) = 6.83; p = 0.018; partial η ^2^ = 0.287), stimulus (F (4, 68) = 36.75; p < 0.0001; partial η ^2^ = 0.684) and ROI (F (1, 17) = 126; p < 0.0001; partial η ^2^ = 0.881). There were also significant two-way interactions between motion and ROI (F (1, 17) = 4; p = 0.031; partial η ^2^ = 0.193), motion and stimulus (F (4, 68) = 5.9; p < 0.0001; partial η ^2^ = 0.26) and ROI and stimulus (F (4, 68) = 17.8; p < 0.0001; partial η ^2^ = 0.51). The three-way interaction between motion, stimulus and ROI was not significant (F (4, 68) = 1.7; p = 0.15; partial η ^2^ = 0.093). To further understand what factors were driving the significant effects we then performed separate two-way ANOVAs on each body-selective ROI.

#### rEBA

A 2 (motion) × 5 (stimulus) repeated-measures ANOVA (Greenhouse-Geisser corrected) showed a main effect of motion (*F* (1, 17) = 14, *p* = 0.001; partial η ^2^ = 0.46) and of stimulus (*F* (4, 68) = 79, *p* < 0.0001; partial η ^2^ = 0.82), with a significantly greater response to bodies than any other stimulus (Bonferroni corrected post-hoc comparisons, all p’s < 0.0001). Motion and stimulus combined in a significant interaction (*F* (4, 68) = 5, *p* = 0.03; partial η ^2^ = 0.235). Post-hoc tests (Bonferroni corrected) showed that moving bodies produced a larger response than static bodies (*p* = 0.01) and that moving objects produced a larger response than static objects (*p* = 0.05). No other post-hoc test approached significance (*p* > 0.1).

#### rFBA

A 2 (motion) × 5 (stimulus) repeated-measures ANOVA (Greenhouse-Geisser corrected) showed a main effect of stimulus (*F* (4, 68) = 59, *p* < 0.0001; partial η ^2^ = 0.8), with a significantly greater response to bodies and to faces than to any other stimulus (Bonferroni corrected post-hoc comparisons, all *p*’s < 0.001). There was no main effect of motion (*F* (1, 17) = 1.4, *p* = 0.25; partial η ^2^ = 0.084) and there was no interaction between motion and stimulus (*F* (4, 68) = 1.5, *p* = 0.2; partial η ^2^ = 0.094).

### Scene-selective ROIs

The neural response to the moving and static face, body, scene, object and scrambled object stimuli in scene-selective ROIs is shown in Fig. 2 (left panels). For scene-selective ROIs a 2 (motion: moving/static) by 5 (stimulus: bodies/faces/objects/scenes/scrambled objects) by 3 (ROI: OPA/PPA/RSC) Greenhouse-Geisser corrected repeated-measures ANOVA found main effects of stimulus (F (4, 68) = 37.7; p < 0.0001; partial η ^2^ = 0.689) and ROI (F (2, 34) = 14.8; p < 0.0001; partial η ^2^ = 0.466) but not of motion (F (1, 17) = 0.3; p = 0.87; partial η ^2^ = 0.002). There were significant two-way interactions between motion and stimulus (F (4, 68) = 7.9; p < 0.0001; partial η ^2^ = 0.316), stimulus and ROI (F (8, 136) = 4.2; p < 0.0001; partial η ^2^ = 0.2) but not between motion and ROI (F (2, 34) = 2.3; p = 0.12; partial η ^2^ = 0.12). The three-way interaction between motion, stimulus and ROI was also significant (F (8, 136) = 4.72; p < 0.0001; partial η ^2^ = 0.217). To further understand what factors were driving the significant effects we then performed separate two-way ANOVAs on each scene-selective ROI.

#### rOPA

A 2 (motion) × 5 (stimulus) repeated-measures ANOVA (Greenhouse-Geisser corrected) showed a main effect of stimulus (*F* (4, 68) = 13, *p* < 0.001; partial η ^2^ = 0.723), with a significantly greater response to scenes than to any other stimulus (Bonferroni corrected post-hoc comparisons, all p’s < 0.001). There was no main effect of motion (*F* (1, 17) = 0.25, *p* = 0.9; partial η ^2^ = 0.088). Motion and stimulus combined in a significant interaction (*F* (4, 68) = 14, *p* < 0.00001; partial η ^2^ = 0.216). Bonferroni corrected post-hoc tests revealed that moving scenes produced a significantly greater response than static scenes (*p* = 0.01).

#### rPPA

A 2 (motion) × 5 (stimulus) repeated-measures ANOVA (Greenhouse-Geisser corrected) showed a main effect of stimulus (*F* (4, 68) = 44, *p* < 0.0001; partial η ^2^ = 0.721), with a significantly greater response to scenes than to any other stimulus (Bonferroni corrected post-hoc comparisons, all *p*’s < 0.001). There was no main effect of motion (*F* (1, 17) = 0.65, *p* = 0.43; partial η ^2^ = 0.037) and no interaction between motion and stimulus (*F* (4, 68) = 0.448, *p* = 0.412; partial η ^2^ = 0.026).

#### rRSC

A 2 (motion) × 5 (stimulus) repeated-measures ANOVA (Greenhouse-Geisser corrected) showed a main effect of stimulus (*F* (4, 68) = 52, *p* < 0.0001; partial η ^2^ = 0.82), with a significantly greater response to scenes than to any other stimulus (Bonferroni corrected post-hoc comparisons, all *p*’s < 0.001). There was no main effect of motion (*F* (1, 17) = 0.8, *p* = 0.4; partial η ^2^ = 0.055) and no interaction between motion and stimulus (*F* (4, 68) = 0.6, *p* = 0.60; partial η ^2^ = 0.035).

## Discussion

Our results show a functional dissociation between category-selective regions located on the lateral brain surface and those located on the ventral and medial brain surfaces. This dissociation was consistent across all three visual categories investigated, suggesting that the networks that selectively process faces, bodies and scenes in the human brain share a common functional organization in response to motion. Lateral areas, including face-selective ROIs in the posterior and anterior superior temporal sulcus (pSTS and aSTS), the body-selective extrastriate body area (EBA) and the scene-selective occipital place area (OPA) all responded more strongly to moving than static stimuli. By contrast, we found no evidence of a difference in the response to moving and static stimuli in ventral and medial category-selective regions, including the face-selective fusiform face area (FFA) and occipital face area (OFA), face-selective voxels in the amygdala, the body-selective fusiform body area (FBA), and the scene-selective retrosplenial complex (RSC) and parahippocampal place area (PPA). Moreover, in face-selective and scene-selective ROIs, this preference for moving, relative to static, stimuli was limited to the preferred stimulus category of the area, i.e., faces in face-selective ROIs and scenes in scene-selective ROIs (Figs 1 and 2). The body-selective EBA, by contrast, showed not only a significantly greater response to moving than static bodies but also a greater response to moving than static objects (Fig. 2). This result is consistent with prior evidence showing the spatial overlap between the EBA and the object-selective lateral occipital complex (LOC) as well as the motion-selective V5/MT^45^.

Prior studies have demonstrated that face-selective ROIs in the STS show a greater response to moving than static faces, while the FFA and OFA show a reduced, or no difference, in the response to moving and static faces^30,38–40^. A similar dissociation between moving and static images of bodies was shown between lateral and ventral areas in a meta-analysis of human movement perception^41^. Most recently, a study of the scene processing network showed that the lateral scene-selective OPA responded more to moving than static scenes, while there was no difference in the response to moving and static scenes in the medial RSC and ventral PPA^42^.

The present study replicates these prior results and extends them in two ways. First, face-selective voxels in the ventromedially located amygdala showed no difference in its response to moving and static faces, thereby demonstrating that the amygdala has the same functional profile as the FFA and OFA (Fig. 1). Second, we simultaneously compared the response to moving and static stimuli in face-, body- and scene-selective areas in the same participants. This design enabled us to demonstrate that a differential response to moving and static stimuli exists in category-selective areas located on the lateral brain surface but is absent in those located on the ventral and medial brain surfaces. This result suggests a common scheme across networks that process different visual object categories. Perhaps this greater response to moving than static stimuli in lateral category-selective areas also extends to lateral brain regions in the human brain that are not category-selective.

There was no difference in the response to moving and static stimuli in the OFA (Fig. 2). This result is consistent with our prior fMRI study that scanned participants using the same experimental stimuli at 3 Tesla^40^. The absence of a difference between moving and static faces is perhaps surprising given that the area is located on the lateral cortical surface in the inferior occipital gyrus^6^. The OFA is thought to process the component parts of a face and is thought to be the earliest face-selective area in the visual cortical hierarchy^1,19^. This has led to the proposal that the OFA selectively processes the primitive, local and stimulus-driven features of a face and should be grouped as a lateral category-selective area together with LOC and the EBA^17^. However, this prior theory did not consider the differential role of motion in the division of category-selective areas.

The broad variety of cognitive operations performed in the STS has led to a debate concerning the functional specificity of the region. One view takes the modular position that different cognitive operations (e.g. face, body and speech perception) are processed in specialized and distinct cortical regions^46^. Another view proposes that the cortical areas encompassing the STS perform a variety of different cognitive operations that are dependent on task-dependent network connections^47^. Our data do not address this debate, but further demonstrate that the lateral regions of occipitotemporal cortex, including the STS, are driven strongly by motion.

The differential response to moving and static stimuli in the pSTS we demonstrated is also consistent with a hypothesis that there are two pathways for face recognition, one inferior and one superior, that begin in early visual cortex^48–51^. The inferior pathway, projecting along the ventral cortical surface, encompasses the OFA and FFA, and is proposed to compute the invariant aspects of a face, such as its identity. The superior pathway, projecting laterally along the STS, is proposed to compute the changeable aspects of a face, including facial expression and direction of eye-gaze. The lack of a significant difference between moving and static faces in both the FFA and OFA also supports this model (Fig. 1). However, in contrast to our data, some prior fMRI studies have reported a higher response to moving than static faces in the FFA^29,52–54^. This discrepancy warrants further investigation but a recent review of the fMRI face processing literature suggested that differences in experimental stimuli could account for the different results^51^. Specifically, the studies reporting a differential response to moving and static faces in the FFA predominately used face morphing software to generate the motion elements in the stimuli. By contrast, prior studies^30,38^ (as well as the current study) that reported no difference between moving and static faces in the FFA used movies of real faces. It is possible that morphed stimuli do not fully capture the changeable aspects of the human face that are apparent in real-world movies^51^.

The most likely source of motion information into the STS (as well as to the EBA and OPA) is the laterally located motion-selective area V5/MT^24^. Neuroanatomical studies in macaques^55,56^ show that V5/MT projects to areas MST and FST, which in turn project to more anterior portions of the STS. A more recent fMRI study in which macaques viewed moving natural stimuli demonstrated that motion, particularly biological motion, accounted for the greatest amount of the neural response in large parts of visual areas, including the STS^57^. In humans, tractography data show a cortical pathway projecting along the lateral surface from occipital cortex, along the STS^49^. Further, our recent combined TMS/fMRI studies show that the response to moving faces in the pSTS and aSTS can be impaired by thetaburst TMS (TBS) delivered over the pSTS^50,58^.

In conclusion, the present study has shown that category-selective regions for faces, bodies and scenes located on the lateral surface of the human brain exhibit a greater response to moving than static stimuli. By contrast, face-, body- and scene-selective regions located on the ventral and medial surfaces exhibit an equal response to moving and static stimuli. This functional dissociation in the response of regions selective for different visual categories, based on brain location, suggests that a response to motion is a common organizing feature in the human brain.

## Methods

### Participants

A total of 22 right-handed participants (13 females) aged between 22 and 46 years old (Mean 27.4 years). All subjects had normal, or corrected-to-normal vision and gave informed written consent before commencing the study. The experimental protocols were approved by the Institutional Review Board (IRB) at the National Institutes of Mental Health (NIMH). All methods, were carried out in accordance with the guidelines and regulations of the NIMH.

### Stimuli

Moving stimuli were 3-second video clips of faces, bodies, scenes, objects and scrambled objects. These stimuli have been used in prior studies^40,50,58–60^. There were sixty video clips for each category. Videos of faces and bodies were filmed on a black background, and framed close-up to reveal only the faces or bodies of 7 children as they danced or played with toys or adults (both of which were out of frame). Face stimuli depicted close-up videos of the child’s face as they performed a range of different actions including; head movement, gaze direction changes, talking (no sound was included) and facial expression changes. Fifteen different locations were used for the scene stimuli, which were mostly pastoral scenes shot from a car window while driving slowly through leafy suburbs, along with some other videos taken while flying through canyons or walking through tunnels that were included for variety. Fifteen different moving objects were selected that minimized any suggestion of animacy of the object itself or of a hidden actor pushing the object; these included mobiles, windup toys, toy planes and tractors, balls rolling down sloped inclines, etc. Scrambled objects were constructed by dividing each object video clip into a 15 by 15 box grid and spatially rearranging the location of each of the resulting video frames. Within each block, stimuli were randomly selected from within the entire set for that stimulus category (faces, bodies, scenes, objects, scrambled objects). This meant that the same video clip could appear within the same block but, given the number of stimuli, this occurred infrequently.

Static stimuli were identical in design to the moving stimuli, except that in place of each 3-second video we presented three different static images taken from the beginning, middle and end of the corresponding video clip. Each image was presented for one second with no inter-stimulus interval, to equate the total presentation time with the corresponding video clip.

### Procedure

Functional data were acquired over 12 blocked-design functional runs lasting 234 seconds each. Each functional run contained two sets of five consecutive stimulus blocks (faces, bodies, scenes, objects or scrambled objects) sandwiched between these rest blocks to make two blocks per stimulus category per run. Each block lasted 18 seconds and contained stimuli from one of the five stimulus categories. The order of stimulus category blocks in each run was palindromic (e.g. fixation, faces, objects, scenes, bodies, scrambled objects, fixation, scrambled objects, bodies, scenes, objects, faces, fixation) and was randomized across runs. For the moving runs, each 18-second block contained six 3-second video clips from that category. For the static runs, each 18-second block contained 18 one-second still snapshots, composed of six triplets of snapshots taken at one-second intervals from the same video clip. Stimuli were presented using Psychtoolbox and Matlab running on a Macbook Pro. Video clips were presented at a frame rate of 70 Hz. Video clips and static stimuli were both presented full screen at a visual angle of 19.8 by 15.7 degrees.

Moving and static runs occurred in the following order: 4 moving, 2 static, 2 moving, 2 static, 2 moving. The first 4 runs of the moving stimuli were used to localize the category-selective regions-of-interest (ROIs) (see ‘Data Analysis’ section). To maintain attention to the stimuli, participants were instructed to press a button when the same stimulus content (e.g. face, body, scene or object) was presented twice in a row (1-back task). On average this occurred once per block. After all functional runs were complete, we collected a high-resolution T-1 weighted anatomical scan to localize the functional activations.

### Brain imaging and analysis

Participants were scanned using a research-dedicated Siemens 7 Tesla Magnetom scanner in the Clinical Research Center on the National Institutes of Health campus (Bethesda, MD). Brain images were acquired using a 32-channel head coil (42 slices, 1.2 × 1.2 × 1.2 mm; 10% interslice gap, TR = 2 s, TE = 27 ms; matrix size, 170 × 170; FOV, 192 mm). Slices were aligned with the anterior/posterior commissure. In addition, a high-resolution T-1 weighted MPRAGE anatomical scan (T1-weighted FLASH, 1 × 1 × 1 mm resolution) was acquired to anatomically localize functional activations. In each scanning session, functional data were acquired over 12 blocked-design functional runs lasting 234 seconds.

Functional MRI data were analyzed using AFNI (http://afni.nimh.nih.gov/afni). Data from the first four TRs from each run were discarded. The remaining images were slice-time corrected and realigned to the third volume of the first functional run and to the corresponding anatomical scan. The volume registered data were spatially smoothed with a 2-mm full-width half-maximum Gaussian kernel. Signal intensity was normalized to the mean signal value within each run and multiplied by 100 so that the data represented percent signal change from the mean signal value before analysis.

A general linear model (GLM) was established by convolving the standard hemodynamic response function with the 5 regressors of interest (one for each stimulus category - faces, bodies, scenes, objects and scrambled objects). Regressors of no interest (e.g., 6 head movement parameters obtained during volume registration and AFNI’s baseline estimates) were also included in this GLM.

The first four moving runs were used to define ROIs using the same statistical threshold (p = 10^−4^, uncorrected) for all participants. We used moving stimuli to localize ROIs because they have been shown to more robustly activate some category-selective regions across participants^30,40^. In addition, our prior work has shown that the pattern of the response across different stimulus categories within a given ROI does not differ when localized with moving stimuli vs. static stimuli^40^.

Face-selective regions were identified using a contrast of activations evoked by moving faces greater than those evoked by moving objects. Body-selective regions were identified using a contrast of activations evoked by moving bodies greater than those evoked by moving objects. Scene-selective regions were identified using a contrast of activations evoked by moving scenes greater than those evoked by moving objects. Within each functionally defined ROI we then calculated the magnitude of response (percent signal change, or PSC, from a fixation baseline) to the moving and static conditions of each of the five stimulus categories (faces, bodies, scenes, objects and scrambled objects), using the data collected from runs 5 to 12 in which pairs of moving and static runs were alternated. All the data used to calculate PSC were independent of the data used to define the ROIs.

## Supplementary information

Supplementary Information
