# Supplementary material for: A functional dissociation of face-, body- and scene-selective brain areas based on their response to moving and static stimuli
Source: Sci Rep. 2019 Jun 3;9:8242. doi: 10.1038/s41598-019-44663-9 (PMC6546694; doi:10.1038/s41598-019-44663-9)
Supplement: Supplementary file 1 — Supplementary Information [file 41598_2019_44663_MOESM1_ESM.pdf]

A functional dissociation of face-, body- and scene-selective brain areas based on their response to moving and static stimuli

David Pitcher [1], Geena Ianni [2,3], & Leslie G Ungerleider [3]

1. Department of Psychology, University of York, Heslington, York, YO105DD, U.K.

2. Weill Cornell / Rockefeller / Sloan Kettering Tri-Institutional MD-PhD Program, New York, 10065, USA.

3. Section on Neurocircuitry, Laboratory of Brain and Cognition, National Institute of Mental Health, Bethesda, MD, 20892, U.S.A.

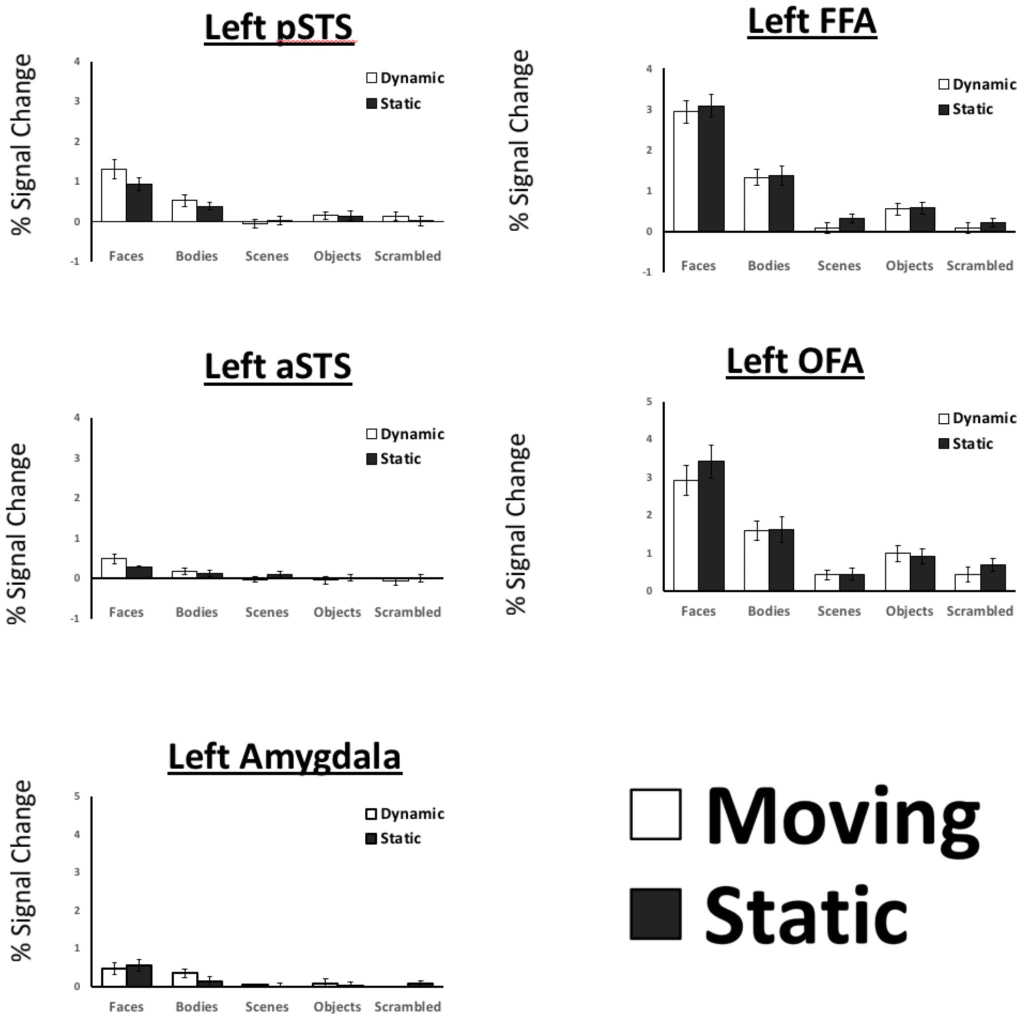

Supplemental Figure 1. Percent signal change data for the moving and static stimuli from all five categories (faces, bodies, scenes, objects and scrambled objects) in face-selective ROIs in the left hemipshere.

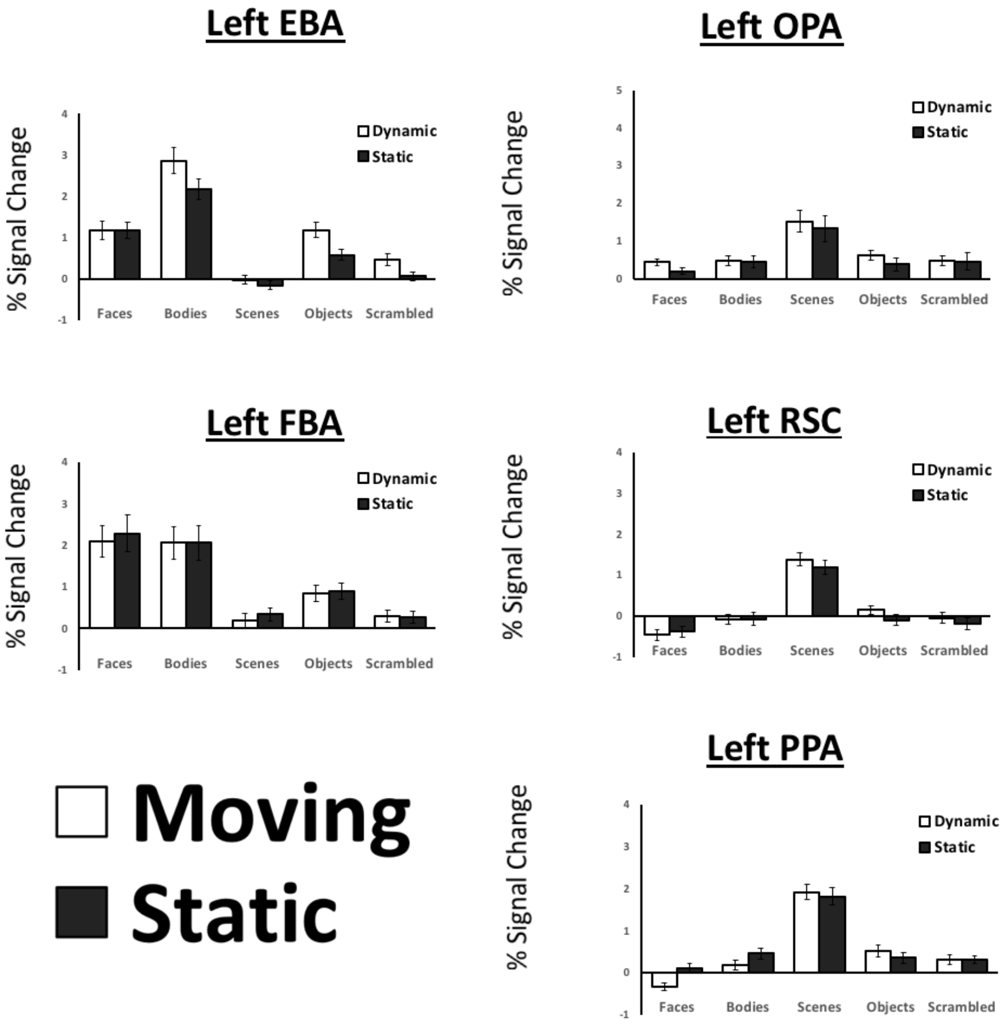

Supplemental Figure 2. Percent signal change data for the moving and static stimuli from all five categories (faces, bodies, scenes, objects and scrambled objects) in body-selective (EBA and FBA) and scene-selective (OPA and RSC) ROIs in the left hemisphere.
